# Advances in the Conceptualization and Measurement of Health Care Empowerment: Development and Validation of the Health Care Empowerment Inventory

**DOI:** 10.1371/journal.pone.0045692

**Published:** 2012-09-19

**Authors:** Mallory O. Johnson, Carol Dawson Rose, Samantha E. Dilworth, Torsten B. Neilands

**Affiliations:** Department of Medicine, University of California San Francisco, San Francisco, California, United States of America; Tehran University of Medical Sciences, Islamic Republic of Iran

## Abstract

The Health Care Empowerment Model offers direction for the investigation of patient-controlled engagement and involvement in health care. At the core of the model is the construct of Health Care Empowerment (HCE), for which there exist no validated measures. A set of 27 candidate self-report survey items was constructed to capture five hypothesized inter-related facets of HCE (informed, engaged, committed, collaborative, and tolerant of uncertainty). The full item set was administered to 644 HIV-infected persons enrolled in three ongoing research studies. Exploratory and confirmatory factor analyses resulted in a two factor solution comprising four items each on two subscales: (1) HCE: Informed, Committed, Collaborative, and Engaged HCE ICCE) and (2) HCE Tolerance of Uncertainty (HCE TU). Subscale scores were evaluated for relationships with relevant constructs measured in the three studies, including depression, provider relationships, medication adherence, and HIV-1 viral load. Findings suggest the utility of this 8-item Health Care Empowerment Inventory (HCEI) in efforts to measure, understand, and track changes in the ways in which individuals engage in health care.

## Introduction

The construct of Health Care Empowerment (HCE) emerged out of a need for a broader conceptualization of engagement in medical treatment that encompasses multiple facets of participation in health care [1]. Specifically, HCE is defined as the process and state of being 1) engaged, 2) informed, 3) collaborative, 4) committed to one’s health care and 5) tolerant or resilient to uncertainties in treatment outcomes. The HCE construct is housed within the larger HCE model, which is informed by multiple theoretical frameworks, including Social Action Theory, Stress and Coping Theory, Social Problem Solving Theory, and multiple derivations of the Health Belief Model [2], [3], [4]. The HCE Model frames a dynamic interplay of contextual factors (e.g., stigma, age, gender), personal resources (e.g., insurance, implicit beliefs about treatment, problem-solving and communication skills), and intrapersonal processes (e.g., depression, anxiety, positive affect, hope) which determine the level of HCE in an individual facing ongoing medical treatment. Because the HCE Model includes a range of hypothesized influences on engagement in medical care, there is potential for studies using an HCE framework to advance our understanding of the drivers of health disparities in such areas as cardiovascular disease, diabetes, and HIV. Consequently, the HCE Model has the potential to inform interventions to mitigate such disparate outcomes in disease incidence and related morbidity and mortality. While there are other constructs and measures in the literature that shed light on factors that may impact engagement in medical care, such as the Patient Activation Measure [5], the newly developed Model of Health Care Empowerment is unique in its focus on multiple, inter-related dimensions of engagement in care that are derived from the patient perspective.

A primary obstacle to further exploration of HCE is a lack of psychometrically sound measures of the construct. There is preliminary evidence that a self-report measure of HCE can shed light on individuals’ health-related beliefs and behaviors [6], but until a strong measurement approach is developed, further work in this area will be limited. We sought to develop a measure of HCE that is aligned with the conceptual framework of the HCE Model. The HCE Model and the newly developed measure were constructed to be applicable across health care contexts, in which complicated psychosocial and environmental factors can impact engagement and outcomes of medical care. In this paper, we describe the development and preliminary validation of a self-report measure of the HCE construct. The development process of the HCE measure was situated within the context of treatment for HIV infection, a setting in which timely and active engagement in care are critical to optimize survival and minimize morbidity.

Active engagement in clinical care and high levels of adherence to antiretroviral treatment (ART) are essential for those with HIV to live longer, healthier lives. As few as 19% of HIV-infected persons in the US have an undetectable viral load. [7] The cascade of missed opportunities for treatment begin with lack of HIV testing and move to lack of linkage to care, retention in care, initiation and adherence to ART. Giordano and colleagues documented that inconsistent engagement and retention in care (as measured by appointment-keeping over time) was associated with death among a large cohort of HIV+ adults [8]. Further, effective ART regimens promote viral suppression, which reduces the chances of HIV transmission to sex partners, and minimizes the emergence of drug-resistant virus. Adherence to ART is difficult due to factors such as the complexity of the regimens, side effects, HIV-related stigma, and competing priorities and demands [9], making HIV treatment a rich setting for the development and evaluation of a new measure of Health Care Empowerment.

The purposes of the current paper are to (a) describe the development of the Health Care Empowerment Inventory (HCEI), a brief, self-report measure of HCE and (b) present evidence of the HCEI’s relationship to variables that are hypothesized correlates as suggested by the HCE Model.

## Methods

### Development of Survey Items

The authors began by generating multiple items to assess each of the descriptors of HCE: engaged, informed, committed, collaborative, and tolerant of uncertainty. Initially, 8–9 items for each descriptor were written for a total of 42 items. A commonly-used 5-level Likert response format was selected in which choices ranged from Strongly Disagree to Strongly Agree. Upon review and feedback from colleagues and experienced research interviewers, items were eliminated for redundancy, lack of clarity, or misalignment with the target construct. Wording on the remaining items was refined to optimize readability. The resulting 27-item set was included in ongoing studies of HIV-infected persons, as described below.

### Sample

A convenience sample of HIV-infected adults 18 years old or older enrolled in one of three research studies was used for the scale development activities. Sample 1 (N = 275) includes data from a longitudinal cohort study of HIV-infected adult men who are in primary relationships with other men (HIV-negative or HIV-positive) collected between January 2009 and April 2012. Sample 2 (N = 370) includes combined cross-sectional data from two research studies of similar samples of HIV-infected adult men and women in the San Francisco Bay Area collected between August 2008 and April 2012. Participants were recruited from community settings through advertisements, flyers, word of mouth referral, and clinic outreach. Data are included from a single time-point in each of the studies (Total N = 645).

### Procedures

Recruitment for the studies included outreach to clinics and agencies, and posting of advertisements and flyers in the San Francisco Bay Area. HIV-positive serostatus (presence of HIV antibodies, indicating HIV infection) was verified by HIV antibody testing or provision of documentation by potential participants, and ART regimens were verified by examination of prescribed medication vials or official medication lists from the dispensing pharmacy. Participants provided written informed consent and all procedures were approved by the Committee on Human Research at the University of California, San Francisco. Combinations of interviewer-administered, Audio Computer-Assisted Self-Interviewing (ACASI) and Computer-Assisted Personal Interviewing (CAPI) were used to optimize self-report, to minimize data collection errors, and to facilitate efficient data management. [10] The HCE items were self-administered in all three studies. Screening, data collection, and phlebotomy procedures occurred in private areas of research facilities or clinics, and all participants were compensated for their involvement.

### Measures

The following measures were administered to the participants in the three studies.

#### Health care empowerment

The 27-item version of the HCE measure, as described above was administered to each study participant.

#### Background and demographic variables

Each study protocol included assessment of participant age, race, ethnicity, education, income, employment status, recent history of injection drug use, length of time since HIV diagnosis, and current use of ART.

### Hypothesized Correlates of HCE

To perform convergent validity analysis, we included the following measures:

#### Adherence self-efficacy

Adherence self-efficacy, or confidence in one’s ability to comply with a treatment plan, has been consistently linked to medication adherence over time. [9], [11] The HIV-ASES scale assesses patient confidence in carrying out health-related behaviors (e.g., asking physician questions, keeping appointments, adhering to medication). [12] This measure includes 2 subscales: Integration and Perseverance; α = .92 and 76 respectively.

#### Opportunity for shared decision-making

The 3-item Decision-Making Opportunity Scale (DMO) [13] assesses how often a provider (a) discusses pros and cons of each medical care choice; (b) elicits statements of patient preference; and (c) takes patient preference into account when making treatment decisions (α = .62). Higher scores indicate a patient’s perception of greater opportunity for involvement as enabled by the provider.

#### Personal knowledge by provider

A single item: “My provider really knows me as a person.” In previous work, higher agreement with this statement was linked to greater ART uptake and adherence [14].

#### Depressive symptoms

The Center for Epidemiologic Studies Depression Scale (CES-D) [15] was administered to measure depressed mood in the past week. The CES-D consists of 20 items rated on a 4-point scale of symptom frequency during the previous week (α = .92).

### Hypothesized Outcomes of HCE

Although the current analyses were restricted to cross-sectional data, we explored associations with variables that are hypothesized consequences of HCE with the recognition that causality cannot be evaluated with the current study data.

#### ART medication adherence

Adherence to antiretroviral medications was assessed using 2 well-validated measures of self-report. The adherence measure developed to assess adherence in the AIDS Clinical Trials Group (ACTG) [16] solicits detailed information about self-reported adherence over the prior 3 days. Adherence scores on this scale have been correlated with viral load. [16], [17] Second, a visual analog scale (VAS) was administered, [18] which assesses 30-day adherence reporting separately for each drug along a continuum anchored by “0%” to “100%.” This measure has shown to be correlated with other adherence measures such as electronic medication monitors. [19], [20] The 3-day adherence measure was dichotomized as 100% vs. <100% adherence and the 30-day VAS adherence is reported as a continuous variable. For both adherence measures, if a respondent was not taking ART but had a CD4 value less than 500, adherence was set to zero as current guidelines indicate that the person should be on ART [21].

#### CD4+ cell count and viral load

HIV viral load was determined using the COBAS® AmpliPrep/COBAS® TaqMan® HIV test kit (Roche Molecular Systems, Inc.), which has a threshold for undetectability ≤20 copies/mL. A detectable viral load indicates incomplete viral suppression, or inadequately controlled HIV infection, and the higher the viral load (transformed to log10 for analysis purposes) the more virus present in the blood with higher numbers indicating poorly controlled HIV. CD4+ cell count provides a gauge of immune functioning, with lower counts typically indicating longer infection and/or greater immune system deterioration. In healthy persons, the normal range of CD4+ cell count is 500–1500 cells per cubic millimeter of blood, and current HIV treatment guidelines recommend ART for all HIV-seropositive persons with CD4+ cell count below 500. [21] For participants in one of the three studies, laboratory assay data were not available. For that subset of respondents (n = 198), we used self-reported CD4 data, which has been shown to be reliable when compared to medical record abstracted data [22].

### Data Analysis

One-way frequency tables were generated to characterize the samples of both studies. Study 1 data were subjected to exploratory factor analysis (EFA) using the WLSMV estimator with geomin rotation in M*plus* 6. The number of factors chosen was based on Cattell’s scree plot, parsimony, and the M*plus* global model fit indices described below [23]. Following exploratory factor analysis of the original 27-item preliminary HCE item set, a reduced number of items were chosen to comprise a brief health care empowerment inventory suitable for use in time-limited settings. This reduced inventory was then subjected to confirmatory factor analysis using M*plus* to assess global model fit of the new inventory [24]. Factor variances were set at unity to set the scale of the latent factors. Satisfactory global factor analysis model fit was determined by two of the following three approximate model fit criteria being met: Bentler’s Comparative Fit Index (CFI) greater than or equal to.95, the root mean square error of approximation (RMSEA) being less than or equal to.06, and the standardized mean square residual (SRMR) being less than.08 in EFA or the weighted root mean square residual being less than 1.00 in CFA [25], [26]. Internal reliability for each subscale in the new HCE instrument was generated using Raykov’s ρ, which is conceptually similar to Cronbach’s coefficient alpha, but relaxes the often unrealistic assumption of equal factor loadings. Confidence intervals for ρ were computed via a logit transformation [27]. Validity and reliability analyses for Sample 2 mirrored those for Study 1, except that validity analyses began with confirmatory factor analyses of the factor-item structure derived in Sample 1.

To assess convergent and divergent validity, Sample 1 and Sample 2 data were pooled and correlations of HCE subscales with available measures of clinical status (e.g., HIV RNA viral load, CD4 T-cell counts) and related behavioral measures (e.g., self-reported self-efficacy for maintaining adherence to antiretroviral medications) were computed with 95% confidence intervals in Stata 12. For multi-category nominal-level correlates (race/ethnicity and education) means of the HCE subscales were compared across groups. To evaluate whether the reduced item subset of the new HCE scale explained a sufficiently substantial proportion of the variance of all items, the sum of all items was regressed onto the new subscales. Due to the clustered dyadic data in Study 1, all confidence intervals and inferences were based on robust Huber-White standard errors that properly account for nesting of individuals within dyads.

## Results

### Sample Characteristics

Characteristics of the sample are provided in Table 1. Sample 1 participants were older and comprised of a lower proportion of African Americans, reported higher levels of education and antiretroviral therapy use, were more likely to be employed, and were less likely to report recent injection drug use. Due to differences in study eligibility criteria, Sample 2 participants included more women, and were more likely to have lower CD4+ cell counts and detectable viral loads.

**Table 1:** Sample Characteristics.

	Sample 1 (*N* = 275) *N* (%)	Sample 2 (*N* = 370) *N* (%)	Total (N = 644) N (%)
Age *m (SD)*	46.92 (9.61)	45.22 (8.15)	45.96 (8.84)
Male	275 (100.00)	283 (78.18)	558 (87.60)
Race	–	–	–
Black/African American	48 (17.45)	166 (45.86)	214 (33.59)
White	145 (52.73)	126 (34.71)	271 (42.54)
Latino	52 (18.91)	42 (11.60)	94 (14.76)
Other	30 (10.91)	28 (7.73)	58 (9.11)
Education	–	–	–
< High School	13 (4.73)	81 (22.25)	94 (14.71)
High School	72 (26.18)	145 (39.84)	217 (33.96)
Some College	76 (27.64)	103 (28.30)	179 (28.01)
College Grad	114 (41.45)	35 (9.62)	149 (23.32)
Working	121 (44.00)	58 (15.57)	178 (27.77)
On Antiretroviral Therapy	258 (93.82)	267 (72.36)	525 (81.52)
Injection Drug Use (past 3 mos)	25 (9.09)	72 (19.89)	97 (15.23)
CD4 Count *m (SD)*	571.89 (253.61)	413.76 (281.03)	481.29 (280.23)
Viral Load Undetectable %	141 (52.03)	146 (40.67)	287 (45.56)
Mos. Since HIV+ *m (SD)*	166.61 (94.03)	147.74 (89.79)	156.04 (92.09)
HCE ICCE *m (SD)*	17.24 (2.08)	16.77 (3.32)	16.97 (2.86)
HCE Tolerance of Uncertainty *m (SD)*	15.39 (2.74)	15.41 (3.94)	15.40 (3.48)

### Sample 1 Validity and Reliability Analyses

The scree plot suggested the presence of two common factors with eigenvalues of 14.12 and 2.46, respectively, in the EFA analysis, which accounted for 61% of the shared variance among the 27 items. All remaining eigenvalues were less than 1.22. As well, global factor analysis model fit statistics indicated satisfactory approximate model-data fit with the two-factor solution: χ^2^(298) = 706.79, *p*<.0001; RMSEA = .07; CFI = .97, and SRMR = .05. Examination of the factor loadings suggested that the first factor was composed of items tapping into the dimensions of being informed, committed, collaborative, and engaged (HCE ICCE). By contrast, the second factor was composed of items measuring tolerance for uncertainty (HCE TU). The two factors were correlated at *r* = .39.

One item each was selected to represent being informed, committed, collaborative, and engaged in an HCE ICCE subscale, whereas four items from the tolerance for uncertainty factor were chosen to represent the HCE TU subscale (Table 2). To assess global model fit of these items to the data, a confirmatory factor analysis was fitted and demonstrated satisfactory approximate model-data fit: χ^2^(19) = 74.15, *p*<.0001; RMSEA = .10; CFI = .96, and WRMR = .97. The factors were correlated at *r* = .42 in the CFA solution. Internal reliability for HCE ICCE (ρ = .78; 95% CI = .73,.83) and HCE TU (ρ = .86; 95% CI = .82,.89) were both within acceptable ranges, suggesting strong internal consistency reliability.

**Table 2:** Factor loadings and 95% Confidence Intervals for Samples 1 and 2.

	Sample 1 (*N* = 275)		Sample 2 (*N* = 369)
Factors and Items	EFA	CFA	CFA
HCE ICCE*			
I prefer to get as much information as possible about treatment options	.72	.64 (.55,.72)	.84 (.79,.88)
I try to get my health care providers to listen to my preferences for my treatment	.73	.69 (.61,.77)	.81 (.76,.86)
I am very active in my health care	.77	.91 (.86,.97)	.87 (.83,.90)
I take my commitment to my treatment seriously	.84	.82 (.76,.89)	.88 (.84,.92)
HCE TU**			
I accept that the future of my health condition is unknown even if I do everything I can	.54	.72 (.64,.80)	.84 (.80,.88)
I recognize that there will likely be setbacks and uncertainty in my health care treatment	.63	.70 (.62,.78)	.86 (.83,.90)
I am comfortable with the idea that there may be setbacks in my treatment	.89	.73 (.66,.81)	.81 (.76,.85)
I have learned to live with the uncertainty of my health condition	.77	.79 (.72,.85)	.85 (.81,.88)

Notes: Items use a 5-point Likert scale from Strongly Disagree to Strongly Agree.

* ICCE = Informed, Committed, Collaborative, Engaged subscale.

** TU = Tolerance of Uncertainty subscale.

### Sample 2 Validity and Reliability Analyses

To fully validate the factor structure generated in Sample 1, it was necessary to fit the final CFA factor structure from Study 1 to a new study’s data, those of Sample 2. A CFA testing the newly-created HCE scale’s factor structure to Study 2′s data demonstrated satisfactory approximate model-data fit: χ^2^(19) = 115.66, *p*<.0001; RMSEA = .12; CFI = .98, and WRMR = .90. The two factors were correlated at *r* = .76 in this study. Internal reliability for HCE ICCE (ρ = .87; 95% CI = .84,.90) and HCE TU (ρ = .90; 95% CI = .88,.92) were also within acceptable ranges, suggesting strong internal consistency reliability.

### Sample 1 and Sample 2 Convergent and Divergent Validity Analyses

The two new HCE subscales accounted for 93% of the variance in the original set of 27 items. Appendix S1 lists the item set for the newly-derived brief measure. Convergent validity analyses showed that HCE ICCE was positively associated with the patient’s perception that the provider knows them as a person, CD4+ cell count, higher ART adherence, adherence self-efficacy, and perceived opportunities for shared decision-making. HCE ICCE was negatively associated with HIV viral load and depressive symptoms (Table 3). HCE TU was positively associated with age, patient perception that the provider knows them as a person, CD4+ cell count, ART adherence, adherence self-efficacy, length of time since HIV diagnosis, and perceived opportunities for shared decision-making. HCE Tolerance was negatively associated with full time employment status and viral load (Table 3). Women reported higher HCE TU than men, and individuals with higher levels of education tended to have higher HCE ICCE scores. Latino participants had a lower mean level of HCE TU than Black and “Other” race/ethnicity participants and participants who were fully employed reported significantly lower mean levels of HCE TU than unemployed participants. Persons on ART reported significantly higher mean levels of both HCE ICCE and HCE TU (Table 3).

**Table 3:** Correlations and Mean Comparisons of HCE ICCE and HCE TU Subscales with Patient Clinical and Attitudinal Variables.

Correlations	N	*r*_HCE_ICCE_ (95% CI)	*r*_HCE_TOL_ (95% CI)
Age	632	.04 (−.03,.11)	.08 (.004,.16)*
Time since HIV Diagnosis	619	.07 (−.01,.14)	.11(.04,.19)*
Depressive symptoms	620_x_	−.16 (−.24, −.08)*	−.08 (−.16,.01)
Provider Knows Me as a Person	616	.26 (.17,.34)*	.22 (.13,.30)*
Decision-making Opportunity Scale	382	.33 (.23,.43)*	.23 (.13,.32)*
Adherence Self-Efficacy Integration	342	.42 (.32,.53)*	.30 (.18,.41)*
Adherence Self-Efficacy Perseverance	341	.31 (.20,.41)*	.31 (.21,.41)*
3-Day Adherence Percentage	435	.28 (.19,.38)*	.14 (.03,.24)*
30-Day Adherence Percentage	614	.32 (.24,.40)*	.18 (.09,.26)*
Detectable VL	626	−.19 (−.26, −.12)*	−.13 (−.20, −.05)*
Log10 Viral Load	626	−.24 (−.32, −.16)*	−.15 (−.23, −.07)*
CD4	632	.20 (.12,.27)*	.06 (−.03,.14)*
**Mean Comparisons**		**Mean (95% CI)**	**Mean (95% CI)**
Gender	634_y_	*F*(1, 538) <0.01, *p* = .98	*F*(1, 537) = 6.67, *p* = .01
Male	556	17.02	15.26
Female	78	17.03	16.47
Race/Ethnicity	638	*F*(3, 538) = 1.10, *p* = .35	*F*(3, 537) = 3.31, *p* = .02
Black/African American	214	16.85 (16.42, 17.28)^a^	15.67 (15.16, 16.17)^a,b^
White	271	17.17 (16.87, 17.48)^a^	15.29 (14.90, 15.67)^a,c,d^
Latino	94	16.64 (16.07, 17.20)^a^	14.54 (13.85, 15.24)^c^
Other	58	17.05 (16.35, 17.76)^a^	16.09 (15.30, 16.88)^b,d^
Education	640	*F*(3, 540) = 8.95, *p*<.001	*F*(3, 539) = 1.00, *p* = .39
Less than High School	94	16.53 (15.81, 17.25)^a,b^	15.90 (15.10, 16.72)^a^
High School Graduate	217	16.46 (16.02, 16.90)^a^	15.13 (14.64, 15.62)^a^
Some College/Technical School	179	17.20 (16.87, 17.54)^b^	15.27 (14.81, 15.73)^a^
College Graduate/Post-Graduate	149	17.76 (17.44, 18.07)	15.50 (15.00, 16.01)^a^
Employed	637	*F*(1, 541) = 0.02, *p* = .88	*F*(1, 541) = 6.43, *p* = .01
No	461	16.99	15.59
Yes	176	16.95	14.82
On ART	640	*F*(1, 544) = 18.69, *p*<.001	*F*(1, 544) = 8.17, *p* = .004
No	118	15.81	14.51
Yes	522	17.26	15.60

Notes: * = *p*<.05. *r*_HCE_ICCE_: correlation of correlate with Health Care Empowerment Informed, Committed, Collaborative, Engaged (ICCE) subscale; *r*_HCE_TOL_: correlation of correlate with Health Care Empowerment Tolerance of Uncertainty (TU) subscale. Means with superscripts are not statistically different at *p*<.05. Subscripts: x *– N* = 621 for *r*_HCE_TU._; *y* – *N* = 633 for HCE Tolerance of Uncertainty (TU) subscale.

## Discussion

The results of the factor analyses and convergent validity analysis suggest that a two-factor structure of HCE fit the data well in the two research samples, and that health care empowerment can be measured with a parsimonious 8-item inventory, comprised of two 4-item subscales, that is suitable for use in time-limited settings. As predicted by the HCE Model, scores on the HCE Inventory (HCEI) were linked in expected directions to depression, provider relationships, treatment adherence, and indicators of clinical status such as CD4 and viral load. The inventory is brief and can be administered in clinical or other community settings and may alert providers to potential suboptimal engagement in care. Because responses were associated with medication adherence, the HCEI may provide an informative signal of adherence problems in contexts where full evaluation of treatment adherence may not be feasible or settings in which nonadherence may be under-reported due to strong social desirability pressures. Scores on both subscales of the HCEI were negatively associated with HIV-1 viral load, suggesting that the higher one’s perception of HCE, the more likely one will have a low or undetectable viral load (i.e. successful treatment and virologic control). Upon future investigation, it may be that HCE scores early in treatment may be a predictive indicator of subsequent poor engagement in care (i.e., failure to attend clinic visits and suboptimal uptake, adherence and persistence with treatment) leading to virologic failure (poor suppression of HIV-1 replication) and higher morbidity and mortality.

Development and initial validation of the HCEI took place within the context of treatment for HIV, but the scale was designed to be applicable across populations and illness contexts. The larger HCE Model accommodates a wide range of illness- and population-specific variables such as disease-related stigma and treatment-specific beliefs. It will be important to establish reliability and validity of the HCEI in other disease contexts in which active engagement in treatment is critical, such as diabetes, cancer, and cardiovascular disease.

There is some indication that empowered patients may be perceived as difficult or demanding by providers [28]. As a result, some patients may temper their assertions in clinical encounters by stifling questions or withholding requests for medical tests, additional opinions, or the pursuit of alternative treatment approaches. The availability of a measure of empowerment in health care allows future investigations into the differential and overlapping roles of patients and providers in establishing health care empowerment. Specifically, the HCEI allows investigation of provider perspectives, reactions, and interactions with patients possessing varying levels of empowerment. Likewise, the HCEI allows investigation of engagement in care across cultures, generations, and genders, where there may be considerable variability in the normative power differentials between patients and providers.

As with any study employing secondary data analysis, findings should be generalized with caution. The studies used convenience samples from one geographic area and relied primarily on self-reported measures such as medication adherence. Data are cross-sectional and thus cannot be used to determine causality and we are not able to evaluate the predictive value of HCEI scores over time. The relatively modest sample sizes and limited variability of gender, age, race, and ethnicity preclude specific analysis of subgroups and findings should thus be considered preliminary. It is also possible that HCEI scores are subject to social desirability reporting biases, which were not assessed in the samples. In spite of these limitations, the resulting brief measure of health care empowerment shows strong psychometric properties, and preliminary validation evidence offers highly promising opportunities for use of the measure in clinical and research settings.

Future work is needed to examine longitudinal patterns of HCEI scores and predictive associations with treatment-seeking, treatment adherence and persistence, and clinical outcomes. Among such outcomes are health-related quality of life, functional impairment, satisfaction with care, and indicators of disease outcomes including morbidity and mortality. The availability of a psychometrically sound measure offers opportunity to investigate potential empowerment-related drivers of disparities in health-related outcomes that exist across populations. Future work is needed to evaluate test-retest reliability of the measure and to identify HCE Model hypothesized determinants and consequences of HCEI scores through associations with other constructs such as personality factors, socioeconomic status, and positive affect over time. Finally, the HCEI provides a device to capture changes in response to interventions designed to increase patient empowerment in health care. In summary, the HCEI offers a useful tool that has the potential to serve as a research and clinical indicator of level of treatment engagement among people living with HIV and other chronic health conditions that require active participation in health care.

## Supporting Information

Appendix S1 **Health Care Empowerment Inventory (HCEI).**
(DOCX)
