# Supplementary material for: Advances in the Conceptualization and Measurement of Health Care Empowerment: Development and Validation of the Health Care Empowerment Inventory
Source: PLoS One. 2012 Sep 19;7(9):e45692. doi: 10.1371/journal.pone.0045692 (PMC3446922; doi:10.1371/journal.pone.0045692)
Supplement: Appendix S1 — Health Care Empowerment Inventory (HCEI). (DOCX) [file pone.0045692.s001.docx]

**Appendix SA: Health Care Empowerment Inventory (HCEI)**

**Instructions:** These questions ask about your involvement in your health care. Please indicate how much you agree or disagree with each of the following statements.

1. I prefer to get as much information as possible about treatment options. (ICCE)
2. I try to get my health care providers to listen to my preferences for my treatment. (ICCE)
3. I am very active in my health care. (ICCE)
4. I take my commitment to my treatment seriously. (ICCE)
5. I accept that the future of my health condition is unknown even if I do everything I can. (TU)
6. I recognize that there will likely be setbacks and uncertainty in my health care treatment. (TU)
7. I am comfortable with the idea that there may be setbacks in my treatment. (TU)
8. I have learned to live with the uncertainty of my health condition. (TU)

**1** = Strongly Disagree

**2** = Disagree

**3** = Neither Agree Nor Disagree

**4** = Agree

**5** = Strongly Agree

Note: ICCE = Informed, Committed, Collaborative, Engaged subscale; TU = Tolerance of Uncertainty subscale
